# *Scn1b* expression in the adult mouse heart modulates Na^+^ influx in myocytes and reveals a mechanistic link between Na^+^ entry and diastolic function

**DOI:** 10.1152/ajpheart.00465.2021

**Published:** 2022-04-08

**Authors:** Daniel O. Cervantes, Emanuele Pizzo, Harshada Ketkar, Sreema P. Parambath, Samantha Tang, Eleonora Cianflone, Antonio Cannata, Govindaiah Vinukonda, Sudhir Jain, Jason T. Jacobson, Marcello Rota

**Affiliations:** ^1^Department of Physiology, New York Medical College, Valhalla, New York; ^2^Department of Pathology, Microbiology and Immunology, New York Medical College, Valhalla, New York; ^3^Molecular and Cellular Cardiology, Department of Medical and Surgical Sciences, Magna Graecia University, Catanzaro, Italy; ^4^School of Cardiovascular Medicine and Sciences, King’s College London British Heart Foundation Centre of Excellence, London, United Kingdom; ^5^Department of Pediatrics, New York Medical College, Valhalla, New York; ^6^Department of Cardiology, Westchester Medical Center, Valhalla, New York

**Keywords:** cell mechanics, diastolic dysfunction, myocytes, voltage-gated sodium channel

## Abstract

Voltage-gated sodium channels (VGSCs) are macromolecular assemblies composed of a number of proteins regulating channel conductance and properties. VGSCs generate Na^+^ current (*I*_Na_) in myocytes and play fundamental roles in excitability and impulse conduction in the heart. Moreover, VGSCs condition mechanical properties of the myocardium, a process that appears to involve the late component of *I*_Na_. Variants in the gene *SCN1B*, encoding the VGSC β1- and β1B-subunits, result in inherited neurological disorders and cardiac arrhythmias. But the precise contributions of β1/β1B-subunits and VGSC integrity to the overall function of the adult heart remain to be clarified. For this purpose, adult mice with cardiac-restricted, inducible deletion of *Scn1b* (conditional knockout, cKO) were studied. Myocytes from cKO mice had increased densities of fast (+20%)- and slow (+140%)-inactivating components of *I*_Na_, with respect to control cells. By echocardiography and invasive hemodynamics, systolic function was preserved in cKO mice, but diastolic properties and ventricular compliance were compromised, with respect to control animals. Importantly, inhibition of late *I*_Na_ with GS967 normalized left ventricular filling pattern and isovolumic relaxation time in cKO mice. At the cellular level, cKO myocytes presented delayed kinetics of Ca^2+^ transients and cell mechanics, defects that were corrected by inhibition of *I*_Na_. Collectively, these results document that VGSC β1/β1B-subunits modulate electrical and mechanical function of the heart by regulating, at least in part, Na^+^ influx in cardiomyocytes.

**NEW & NOTEWORTHY** We have investigated the consequences of deletion of *Scn1b*, the gene encoding voltage-gated sodium channel β1-subunits, on myocyte and cardiac function. Our findings support the notion that *Scn1b* expression controls properties of Na^+^ influx and Ca^2+^ cycling in cardiomyocytes affecting the modality of cell contraction and relaxation. These effects at the cellular level condition electrical recovery and diastolic function in vivo, substantiating the multifunctional role of β1-subunits in the physiology of the heart.

## INTRODUCTION

Voltage-gated sodium channels (VGSCs) are macromolecular complexes primarily composed of one pore-forming α-subunit and one- or two-transmembrane β-subunits, known to alter gating, voltage dependency, and kinetics of the channel (1). The five nonpore-forming β-subunits found in mammals also act as cell adhesion molecules, playing roles in neuronal proliferation and migration during brain development (2) as well as cell-to-cell interaction molecules at the level of the cardiac intercalated disk (3). Emerging evidence also supports the notion that VGSC β1-subunits modulate gene expression in heart via a process that appears to involve proteolytic cleavage of the β1-protein and generation of a soluble intracellular domain, which translocates to the nucleus, where it acts as transcriptional regulator (4).

VGSC β1/β1B-subunits are expressed in the nervous system and heart and variants of *SCN1B*, encoding the β1- and β1B-subunits, are associated with mild to severe forms of epilepsy and cardiac arrhythmias (2). These debilitating conditions are consistent with aberrant cell excitability secondary to altered VGSC conductance. However, structural abnormalities originating from pathological cell migration and/or altered gene expression may also contribute, in part, to manifestations of these channelopathies (2). Thus, understanding canonical and noncanonical functions of VGSC β1/β1B-subunits may provide critical information on the role of these proteins in normal, physiological conditions, and their contribution to the origin and progression of diseased states.

Experimentally, the expression of *SCN1B* variants from patients with proarrhythmic behavior in heterologous cell systems (5, 6) or cardiomyocytes derived from human induced pluripotent stem cells (7) resulted in Na^+^ current perturbations, substantiating the crucial role of β1- and the β1B-subunits in the electrical behavior of cardiac cells. These in vitro findings have been corroborated by results from juvenile *Scn1b*-null mice, in which loss of β1-subunits led to potentiated transient and late Na^+^ current in ventricular myocytes (4, 8, 9) and to prolongation of the QT interval of the electrocardiogram, in vivo (9). In addition, cardiac-restricted and constitutively active loss of *Scn1b* in adult mice were found to prolong the duration of the action potential (AP) and to disrupt the intracellular Ca^2+^ homeostasis of myocytes (8). The defective Ca^2+^ cycling was normalized by inhibition of the tetrodotoxin-sensitive Na^+^ current and appeared to contribute to the enhanced susceptibility of *Scn1b*-null hearts to ventricular arrhythmias (8). Thus, β1/β1B-subunits are integral components of the VGSC macromolecular complex and, by modulating Na^+^ current, have important consequences on myocytes and cardiac electrophysiology.

In addition to the channelopathies linked to mutations of proteins of the VGSC macromolecular complex (10), posttranslational modifications targeting the channel assembly are key modifiers of Na^+^ current properties in cardiomyocytes, under both physiological and diseased conditions (11, 12). Importantly, the possibility has been raised that consequences of altered Na^+^ fluxes are not restricted to the electrical stability of the heart, but also contribute to mechanical dysfunction of the myocardium, via dysregulation of intracellular ionic homeostasis (13, 14). Increased Na^+^ influx raises the intracellular Na^+^ concentration ([Na^+^]_i_), which interferes with diastolic Ca^2+^ extrusion via the Na^+^/Ca^2+^ exchanger (NCX), enhancing cytoplasmic Ca^2+^ load (13–15) and affecting myocyte mechanics. In this regard, the late Na^+^ current (*I*_NaL_) is enhanced in inherited and acquired conditions (16–18) and has emerged as a potential therapeutic drug target for cardiovascular disorders (17, 18). *I*_NaL_ inhibition in cells and tissue from human diseased hearts attenuated diastolic Ca^2+^ and passive tension, respectively (16, 19), partly justifying improvements of diastolic indices seen in patients treated with inhibitors of *I*_NaL_ (20–22). Thus, although it is clear that VGSCs modulate myocyte and cardiac electrical behavior, whether integrity of the channel assembly is required to preserve the mechanical function of the adult heart remains to be clarified.

In this study, we examined the role of *Scn1b* on cellular and cardiac electromechanical function by using adult engineered mice with cardiac-restricted, inducible deletion of *Scn1b*. The inducible, cardiac-restricted model of *Scn1b* deletion was implemented to circumvent seizures and lethality of offspring associated with complete loss of *Scn1b* gene (23) and to evaluate the function of β1/β1B-subunits on the physiology of the adult heart, without the potential consequences associated with loss of *Scn1b* gene during development and early life (4, 9). We report that loss of *Scn1b* in adult cardiomyocytes enhances Na^+^ current, alters properties of Ca^2+^ transients, delays kinetics of contraction and relaxation, and impairs diastolic function in vivo. These defects, occurring in the absence of structural modifications of the heart, are normalized following *I*_NaL_ inhibition, strengthening the causative link between enhanced Na^+^ influx and manifestation of diastolic dysfunction. Overall, these findings support the key role of VGSC integrity in the physiology of the heart.

## METHODS

All data, materials, and methods of this study are available from the corresponding author upon reasonable request.

### Animals

Mice were maintained in accordance with the Guide for Care and Use of Laboratory Animals; animal experiments were approved by the local animal care committees (IACUC) of New York Medical College. When needed, isoflurane (1%–1.5%, inhalation) was employed as a methodology of anesthesia. Euthanasia was attained under anesthesia by bilateral thoracotomy and removal of the heart.

To obtain mice with conditional and inducible deletion of *Scn1b* in myocytes, a *Scn1b^flox/flox^*/αMHC-MerCreMer (conditional knockout, cKO) mouse line was obtained by intercrossing *Scn1b^flox/flox^* animals congenic on the C57Bl/6J background (4, 8, 9) with B6.FVB(129)-A1cf^Tg(Myh6-cre/Esr1^*^)1Jmk^/J mice expressing tamoxifen-inducible Cre recombinase under the α-myosin heavy chain promoter (αMHC-MerCreMer, Jackson Labs, Stock No. 005657) (24, 25), backcrossed onto C57BL/6J by the vendor. Double transgenic mice homozygous for the floxed allele and homozygous for MerCreMer construct were employed. *Scn1b^flox/flox^* mice were developed and kindly donated by the laboratory of Dr. Isom, at the University of Michigan.

Cre recombinase was induced by administration of tamoxifen (Sigma-Aldrich) dissolved in 10% ethanol and 90% peanut oil (Sigma-Aldrich) for 4 consecutive days (30 mg/kg of body wt/day ip) (24, 26). The inducible, cardiac-restricted model of *Scn1b* (cKO) deletion was used to circumvent seizures and lethality of offspring associated with complete loss of *Scn1b* gene (23) and to avoid the effects of enhanced Na^+^ current in ventricular cardiomyocytes during development (9). Single transgenic mice homozygous for MerCreMer construct were treated with tamoxifen and employed as control (conditional MerCreMer, cMCM). The use of the inducible MerCreMer system required careful consideration of the experimental protocol to be employed, to minimize off-target and confounding effects of tamoxifen administration and Cre recombinase expression in the heart (26, 27). Based on the survival of cMCM and cKO cohorts of mice, which was comparable and not affected after ∼20 days following tamoxifen administration (Supplemental Fig. S1; all Supplemental Material is available at https://doi.org/10.6084/m9.figshare.19326329), studies were restricted to mice monitored for 4 wk or more after induction of genomic recombination. For survival tests, the same group of animals was considered before (baseline) and after tamoxifen treatment, to induce cardiomyocyte-restricted Cre expression alone (cMCM) or in combination with *Scn1b* gene deletion (cKO). At euthanization, median age of mice and median time after tamoxifen treatment were 4.8 mo (3.5–7.0 mo) and 49 days (28–94 days), respectively. Unless otherwise specified, collected data were disaggregated by sex. A cohort of male C57Bl/6N mice at ∼3 mo of age was obtained from Charles River and employed for ex vivo studies.

### In Vivo Cardiac Function

Echocardiography was performed in conscious mice using Acuson Sequoia c512 equipped with a 13-MHz (15L8) linear transducer (28–30). By this approach, parasternal short axis view of the left ventricle (LV) was employed to evaluate the chamber diameter and wall thickness in diastole and systole, for the computation of LV volume, mass, and ejection fraction (EF) (28–31). Diastolic function was assessed using pulsed-wave Doppler imaging of the transmitral filling pattern in the apical four-chamber view of the heart (28, 29). Early transmitral filling wave (E wave) and late filling wave due to atrial contraction (A wave) were obtained. Isovolumic relaxation time was calculated as the time from closure of the aortic valve to the initiation of the E wave (28, 29).

Electrocardiograms (ECGs) were recorded under isoflurane anesthesia by inserting needle electrodes subcutaneously into the mouse limbs (28, 30). Electrical signals were amplified with a 12-Lead ECG Amplifier (DSI, Ponemah), digitized using a 160-kHz A/D converter (DI-1120 HS, Dataq), and recorded with WinDaq software (Dataq). Surface ECG intervals were measured using LabChart8. Spontaneous cycle length was determined by averaging 25 consecutive R-R intervals. PR interval, QRS duration, and QT interval were measured by determining the earliest onset and latest offset of atrial and ventricular deflections from the averaged cycles (28–30).

To record ECGs in conscious animals, an ECG-tunnel device (Emka Technologies) was employed (32). Animals were placed in a tunnel and ECGs recorded for a period of 10 min. Electrical signals were amplified with a 12-lead ECG amplifier (DSI, Ponemah), digitized using a 160-kHz A/D converter (DI-1120 HS, Dataq), and recorded with WinDaq software (Dataq). Electrical signals were evaluated offline with LabChart8 for the occurrence of rhythm disturbances (32).

Left ventricular (LV) hemodynamics and pressure-volume (PV) loops were obtained in anesthetized mice (isoflurane, 1.5%) in the closed chest preparation with a MPVS Ultra system for small animals (Millar Instruments) equipped with a SPR-839 catheter and coupled to a PowerLab/8SP A/D converter (ADInstruments) (28–30). The mouse was warmed with a heat lamp; the right carotid artery was exposed, and the pressure transducer was inserted and advanced in the LV cavity. Data were acquired with LabChart7 (ADInstruments) software. Baseline PV loops and loops following inferior vena cava occlusion were collected to compute the slope of end-diastolic PV relations (EDPVR), which is an indicator of LV stiffness (29, 33). PV loops free of respiratory motion artifacts were employed for analysis. Inferior vena cava occlusion was achieved by compression, with a cotton tip applicator, of the inferior vena cava accessed immediately below the diaphragm via a small abdominal incision (29, 34). For calibration of SPR-839 catheter and evaluation of LV blood volume, manufacturer’s instructions were followed. Briefly, cuvette calibration protocol with fresh heparinized, warm blood, and in vivo bolus infusion of hypertonic saline solution (15% NaCl) was performed to compute slope and intercept and to assess parallel conductance. Analysis was performed using LabChart software.

In vivo blockade of the late Na^+^ current (*I*_NaL_) was achieved by the administration of GS967 (Apexbio, 0.5 mg/kg body wt ip) (32, 35, 36) or mexiletine (Sigma-Aldrich, 5 mg/kg body weight, ip) (29, 37) dissolved in USP saline solution. Effects of *I*_NaL_ inhibition were tested at ∼30–100 min after drug administration in animals at ≥4 wk after induction of gene recombination.

### Ex Vivo Properties of the Mouse Heart

For ex vivo studies, the ascending aorta was cannulated with PE-50 tubing connected to a 23-G three-quarter needle. Subsequently, hearts were perfused in a Langendorff apparatus (Radnoti). Perfusion was accomplished at a constant pressure of ∼80 mmHg with prewarmed Krebs–Henseleit buffer (KHB; Sigma-Aldrich), containing (in mmol/L) 118 NaCl, 4.7 KCl, 11 glucose, 1.2 MgSO_4_, 1.2 KH_2_PO_4_, 1.8 CaCl_2_, and 25 NaHCO_3_, gassed with 95% O_2_ and 5% CO_2_ (pH 7.4) at 37°C (28–30, 38, 39). The temperature was maintained by immersing the heart in a water-heated glassware reservoir (Radnoti), containing preheated KHB. Hearts were stimulated at 8 Hz (125-ms cycle length) with a 3-ms square pulse at 1.5–2-fold its threshold level (4 channels stimulator, BMS 414, Crescent Electronics; S48, Grass; or ISO-STIM 01 M, npi), using a mini-coaxial electrode (Harvard Apparatus).

To assess electrical activity of perfused hearts, monophasic action potentials (MAPs) were recorded using a micro-MAP-tip electrode (Harvard Apparatus) positioned on the LV-free wall (28, 29, 38, 39). A two-lead mini-ECG system (Harvard Apparatus), in which electrodes were placed on the right atrium and apex of the heart, was used to obtain pseudo-ECG (29, 30, 39). Electrical signals were amplified (6600 Amplifier, Gould Instruments), digitized using a 160-kHz A/D converter (DI-1120 HS, Dataq), and recorded with WinDaq software (Dataq).

Programmed electrical stimulation (PES) was introduced to assess the propensity of the mouse heart to develop ventricular arrhythmias (29, 39). A stimulus generator (STG2004, Multi Channel System) controlled by a PC was coupled with a stimulus isolation unit (ISO-STIM 01 M, npi) to apply PES protocols. Initially, a train of 20 pacing stimuli (S1) applied at 125 ms cycle length with an extra stimulus (S2) inserted at the end of the train was employed. The S1–S2 interval was progressively reduced until the S2 stimulus either failed to generate an action potential or induced ectopic events (29). A second protocol consisted of a 10-s stimulation train at constant frequency followed by a 10-s rest period. Trains of progressive higher frequency (5–22 Hz) were applied till ectopic events occurred or stimulation of 22 Hz was reached. The appearance of premature ventricular complexes (PVCs, ectopic beats characterized by atria-ventricular dissociation), ventricular tachycardia (three or more consecutive ectopic beats), and/or ventricular fibrillation was established (29, 39). Data were analyzed with LabChart8 software.

Isometric LV pressure was measured ex vivo using a fluid-filled balloon catheter connected to a pressure transducer (Harvard Apparatus) (30). Pressure signal was amplified (Bridge Amp, ADInstruments), digitized using a 4-kHz A/D converter (Power Lab 8/30, ADInstruments), and recorded using LabChart7 software. Pseudo-ECGs were also obtained. The fluid-filled balloon, made of a small square of polyethylene film, was inserted into the LV. Following an equilibration period, diastolic pressure-volume relationships were established by increasing the LV balloon volume in ∼2-µL increments with an air-tight syringe. The LV volume was increased up to a value (V_max_) at which peak LV developed pressure was reached and a further increase in balloon volume led to a decrease in peak pressure. Data were analyzed with LabChart software.

The late Na^+^ current was enhanced with anemonia toxin II (ATX-II, 5 nmol/L, Sigma-Aldrich) dissolved in KHB (29, 40, 41).

The LV pressure-volume relation was established using ex vivo assays and fluid-filled balloon catheter (29, 30). Following the measurement of the volume required to reach maximal developed pressure (V_max_), diastolic pressure-volume relations were plotted in the range of volumes comprised between *V*_1/2_ (half of V_max_) and V_max_. LV diastolic pressure was offset at *V*_1/2_. Relations were fitted with a second-order polynomial equation:

(1) $${\text{dP}}_{\left(\text{V}/\text{Vmax}\right)}=a\cdot \left(\text{V}/{\text{V}}_{\text{max}}\right)\;+b\cdot \;{\left(\text{V}/{\text{V}}_{\text{max}}\right)}^{\text{2}},$$

where dP is the diastolic pressure and V is the volume of the LV (29).

### Isolation of Adult Ventricular Cardiomyocytes

With the animal under deep anesthesia (isoflurane) and following administration of heparin (∼200 U ip), thoracotomy was performed, heart was excised, and left ventricular (LV) myocytes were enzymatically dissociated as previously reported (24, 28–30, 38, 39, 42, 43). Briefly, the heart was connected to a plastic cannula for retrograde perfusion through the aorta in a Langendorff system (Radnoti), at 37°C. Perfusate consisted of a Ca^2+^-free solution gassed with 100% O_2_. After 5 min, 0.1 mmol/L CaCl_2_, 274 U/mL collagenase (type 2, Worthington Biochemical), and 0.57 U/mL protease (Type XIV, Sigma-Aldrich) were added to the solution, which contained (in mmol/L) 126 NaCl, 4.4 KCl, 5 MgCl_2_, 20 HEPES, 22 glucose, 20 taurine, 5 creatine, 5 Na pyruvate, and 5 NaH_2_PO_4_ (pH 7.4). At completion of digestion, atria and right ventricle were dissected and discarded. Remaining LV-free wall and septum were separated. Tissue cut in small pieces and these fragments were shaken in resuspension solution and filtered using a 230-µm sieve (Sigma-Aldrich). For physiological studies, only rod-shaped LV-free wall myocytes exhibiting cross striations and showing no spontaneous contractions or contractures were selected. Cells were used within 8 h following enzymatic digestion.

### Whole Cell Patch-Clamp Studies

Isolated adult LV myocytes were placed in a bath on the stage of a Zeiss Axiovert 200 microscope for patch-clamp measurements (24, 28, 29, 39, 41, 44). Data were acquired by means of the whole cell patch-clamp technique in voltage- and current-clamp modes using a multiclamp 700B amplifier (Molecular Devices). Electrical signals were digitized using a 250-kHz 16-bit resolution A/D converter (Digidata 1550B, Molecular Devices) and recorded using pCLAMP 10 software (Molecular Devices) with low-pass filtering at 2 kHz. Membrane capacitance (*C*_m_) was measured in voltage-clamp mode using a 5-mV voltage step and pCLAMP software algorithm; this parameter was employed to normalize transmembrane currents (24, 29, 39). Pipettes were pulled by means of a horizontal (P-97, Sutter Instruments) glass microelectrode puller; when filled with intracellular solution, pipettes had a resistance of 1–3 MΩ.

For action potential (AP) measurements, cells were stimulated with current pulses 1–1.5-fold threshold (24, 28–30, 39, 41, 44). Myocytes were bathed with Tyrode solution containing (in mmol/L) 140 NaCl, 5.4 KCl, 1 MgCl_2_, 5 HEPES, 5.5 glucose, and 1 CaCl_2_ (pH 7.4, adjusted with NaOH). The composition of the pipette solution was (in mmol/L): 10 NaCl, 15 KCl, 100 K-aspartate, 0.5 MgCl_2_, 5 K_2_-ATP, 5.5 glucose, 5 CaCl_2_, 10 EGTA, and 10 HEPES (pH 7.2 with KOH). The combination CaCl_2_ and EGTA resulted in [Ca^2+^]_i_ buffered at ∼150 nmol/L (39, 45). Recorded transmembrane potentials were corrected for the liquid junction potential, which was calculated using the Junction Potential tool of Clampex (pCLAMP). AP measurements were conducted at 37°C. Data were analyzed using Clampfit (pCLAMP) software.

Transient, or fast, Na^+^ current (*I*_Na_) was measured in adult ventricular myocytes with a previously reported protocol (24, 29). Cells were bathed at room temperature with a modified Tyrode solution of the following composition (in mmol/L) 5 NaCl, 1 MgCl_2_, 1 CaCl_2_, 0.1 CdCl_2_, 20 HEPES, 11 glucose, and 132.5 CsCl (pH 7.4 with CsOH). The composition of the pipette solution was (in mmol/L) 5 NaCl, 135 CsF, 10 EGTA, 5 Mg-ATP, and HEPES 5 (pH 7.2 with CsOH). Following *C*_m_ and series resistance compensation (70%), *I-V* relations were determined applying depolarizing steps 200 ms in duration from holding potential (*V*_h_) −120 mV in 5-mV increments. Interpulse interval was 3 s. *I*_Na_ amplitude was measured as the current difference between the inward peak current and the current at the end of the 200-ms step. *I*_Na_ was normalized by *C*_m_.

At each potential tested (*V*_m_), Na^+^ conductance (*g*) was calculated as

(2) $$g=I_{\text{Na}}/\left(V_{\text{m}}-E_{\text{Na}}\right),$$

where *I*_Na_ is the amplitude of the Na^+^ current at *V*_m_ and *E*_Na_ is the reversal potential, which was defined by the intercept of the *I-V* relation to the zero-current axis.

Conductance-voltage relations were plotted and fitted with the following Boltzmann function:

(3) $$g=g_{\text{max}}/\left\{\text{1 }+\text{ exp}\left[\left(V_{\text{1}/\text{2}G}-V_{\text{m}}\right)/k_{G}\right]\right\},$$

where *g_max_* corresponds to maximal conductance, *V*_1/2_*_G_* is the potential at which conductance is halfway between 0 and *g*_max_, and k*_G_* is the slope of the conductance curve.

Values of normalized Na^+^ conductance (*G* = *g*/*g*_max_) were plotted to obtain activation curves; half maximal activation potential (*V*_1/2_*_G_*) and slope of the activation curve (*k_G_*) were obtained by fitting the data with a Boltzmann equation:

(4) $$G=\;{\left\{\text{1 }+\text{ exp}\left[\left(V_{\text{1}/\text{2}G}-V_{\text{m}}\right)/k_{G}\right]\right\}}^{-\text{1}}.$$

To dissect fast (*I*_Na,fast_) and slow components (*I*_Na,slow_) of *I*_Na_, the decay phase of the inward current at its maximum peak was analyzed with a biexponential function. Currents were normalized by *C*_m_ and expressed as pA/pF.

To evaluate Na^+^ influx in myocytes, time integral of *I*_Na_ was calculated during the 50 ms that followed the inward peak of *I*_Na_ at its maximum amplitude. Current integral was normalized by *C*_m_.

A two-pulse protocol was used to assess the voltage dependence of steady-state inactivation of *I*_Na_ (24, 29). Prepulses 300 ms in duration were introduced to depolarize the cell to different membrane voltages starting from −120 mV to −35 mV, in 5-mV increments. Each prepulse was followed by a single 30-ms test pulse, which depolarized the cell to −40 mV. Values of normalized Na^+^ conductance (*G* = *g*/*g*_max_) were plotted with *V*_m_ relative to the preconditioning steps to compute the steady-state inactivation curves, which were fitted with a Boltzmann equation, as indicated above in *Eq*. *4*.

*I*_Na_ reactivation was studied using a two-pulse protocol with variable interpulse duration (24, 29), ranging from 1 ms to 50 ms, in increments of 1 ms. With each pulse, *I*_Na_ was activated by depolarizing cells from *V*_h_ −120 mV to −40 mV for 30 ms. The double pulse protocol was repeated every 3 s. For each interpulse duration (t), *I*_Na_ reactivation was calculated by dividing the amplitude of the current measured during the second pulse (*I*_t_) by the amplitude of the current measured during the first pulse with maximal amplitude (*I*_max_). Values of *I*_Na_ reactivation were plotted to obtain reactivation curves. Reactivation relations were plotted and fitted with the following two-phase association equation:

(5) $$I_{\text{t}}/I_{\text{max}}=I_{\text{fast}}\cdot \left[\text{1 }-\text{ exp}\left(-{\text{k}}_{\text{fast}}\cdot t\right)\right]\;+I_{\text{slow}}\cdot \left[\text{1 }-\text{ exp}\left(-{\text{k}}_{\text{slow}}\cdot t\right)\right],$$

where *I*_fast_ and *I*_slow_ are the fractions of *I*_max_ accounted for by the fast and slow reactivating components of *I*_Na_; k_fast_ and k_slow_ are the two rate constants, expressed in reciprocal of ms.

### Cell Shortening

Acutely isolated LV myocytes were placed in a perfusion chamber with field stimulation (Warner Instruments) on the stage of an inverted microscope (Swift) for contractility measurements (29, 39, 41, 43). Cells were bathed continuously with Tyrode solution warmed at 37°C. Measurements were collected in field-stimulated cells by a CCD camera (TM-540, Pulnix), video edge detection (VED-205, Crescent Electronics), a 10-kHz A/D converter (DI-155 HS, Dataq), and WinDaq software (Dataq). Contractions were elicited at 1 Hz by rectangular depolarizing pulses, 3 ms in duration and ∼1.5-fold threshold in intensity, with platinum electrodes connected to a stimulator (SD9, Grass). Unless otherwise specified, for each animal, 40–60 cells were employed to characterize the contractile behavior of cardiomyocytes for the two mouse genotypes. To test the effects of increased pacing rates, myocytes were stimulated at 1 Hz and 4 Hz. The late Na^+^ current (*I*_NaL_) was blocked with 300 nmol/L GS967 (Apexbio) (46, 47) or with 10 µmol/L mexiletine (Sigma-Aldrich) (29, 41). Parameters of cell shortening and relaxation were analyzed using LabChart8 software.

### Ca^2+^ Transients

Acutely isolated LV myocytes were placed in a perfusion chamber with field stimulation (Warner Instruments) on the stage of an upright microscope (BX61WI, Olympus) for the evaluation of Ca^2+^ transients (29, 30, 39, 42). Cells were bathed continuously with Tyrode solution warmed at 37°C. Measurements were collected in field-stimulated cells by IonOptix fluorescence systems (IonOptix) connected to a 10-kHz A/D converter (DI-155 HS, Dataq). Signals were acquired using WinDaq software (Dataq). Ca^2+^ transients were elicited at 1 Hz by rectangular depolarizing pulses, 3 ms in duration, and ∼1.5-fold threshold in intensity, with platinum electrodes connected to a stimulator (SD9, Grass). Unless otherwise specified, for each animal, 40–60 cells were employed to characterize Ca^2+^ transient properties of cardiomyocytes for the two mouse genotypes. To test the effects of increased pacing rates, myocytes were stimulated at 1 Hz and 4 Hz. Ca^2+^ transients were assessed by epifluorescence after loading myocytes with 0.5 µmol/L Fluo-4 AM (Invitrogen). Excitation wavelength was 480 nm with emission collected at 535 nm using a ×60, long distance, water immersion objective (LUMPlanFI, Olympus). Fluorescence excitation was restricted to the central area of the cell using a field diaphragm, whose aperture was maintained constant. Parameters of Ca^2+^ transients were analyzed using LabChart8. Fluo-4 signals were expressed as fluorescence intensity (Fluo). The late Na^+^ current (*I*_NaL_) was blocked with 300 nmol/L GS967 (46, 47) or with 10 µmol/L mexiletine (29, 41).

### Myocyte Size

Images of LV myocytes fixed in paraformaldehyde were collected with an inverted microscope (CK2, Olympus) equipped with a digital camera (MU1003, Amscope). Cell length and area were evaluated using ImageJ software. Average cell width was calculated by dividing cell area by cell length (long axis). Cell volume was then computed assuming the shape of myocytes as a flattened cylinder with elliptical cross section (29, 41), in which the major axis corresponds to the average cell width and the ratio major-to-minor axis is 1.8, based on previous three-dimensional reconstructions for mouse cardiomyocytes (29).

### Histological Analysis

Mouse hearts were perfused and stored in neutral buffered, 10% formalin solution (Sigma-Aldrich) for histological analysis. Transversal sections of the LV at the midventricular level were embedded in paraffin and sliced to obtain thin sections (∼4 mm thickness) (29, 48). For detection of connective tissue, slides were trichrome-stained (Masson’s Trichrome Stain Kit, Mastertech, StatLab) following manufacturer’s instruction. Images were acquired using an inverted microscope (CK40, Olympus) with ×20 objective equipped with a digital color camera (MU1003, Amscope). Interstitial fibrosis was quantified with respect to total tissue area using ImageJ software (29, 41).

### Quantitative RT-PCR

Total cellular RNA was prepared from mouse LV isolated cardiomyocytes using RNeasy Mini kit (Qiagen) following manufacturer’s instructions. cDNA was obtained from total RNA using Transcriptor High Fidelity cDNA Synthesis kit (Sigma-Aldrich). Real-time RT-PCR was performed with primers listed in Supplemental Table S1. The QuantStudio Real-Time PCR system (Applied Biosystems) was employed for quantitative RT-PCR. In each case, cDNA was combined with Fast SYBR Green Master Mix (Applied Biosystems) in a 25-μL reaction mix. Cycling conditions were as follows: 95°C for 10 min followed by 40 cycles of amplification (95°C denaturation for 10–15 s, 60°C annealing and extension for 1 min). The melting curve was then obtained. C_t_ values were normalized with respect to hypoxanthine guanine phosphoribosyl transferase (*Hprt*).

### Western Blot Analysis

Whole protein extracts from snap-frozen LV myocardium were prepared using RIPA Lysis and Extraction Buffer (Thermo Fisher Scientific), supplemented with a Protease and Phosphatase Inhibitor Cocktail (Sigma-Aldrich). Equivalent of 50 µg of proteins were prepared with SDS Sample Buffer (Sigma-Aldrich), separated on either 4%–15% or 7.5% SDS-PAGE (Mini-PROTEAN TGX Stain-Free Protein Gels, Bio-Rad) with Tris-glycine-SDS (Bio-Rad). High molecular weight proteins were separated on 7.5% gels for 4 h on ice, at constant voltage (90 V). Proteins were transferred onto PVDF membrane (Li-Cor) with Tris-glycine transfer buffer (Bio-Rad) containing 20% or 5% (for high molecular weight proteins) methanol. Subsequently, total protein load was quantified using a protein stain (Revert 700, Li-Cor). Membranes were washed with Tris-buffered saline (TBS, Bio-Rad) with 0.1% Tween 20 (Thermo Fisher Scientific) blocked with intercept blocking buffer (Lic-cor), for 1 h at room temperature. Membranes were incubated overnight at 4°C with primary antibodies listed in Supplemental Table S2, at the indicated dilution. Fluorescent-labeled secondary antibodies (IRDye 800CW or 680LT Secondary Antibodies) were used for signal detection with an infrared imaging system (Odyssey CLx, Li-Cor). Western blot analysis protocols with various antibodies were optimized before quantitative analysis. For molecular weight identification, Chameleon kit Prestained Protein Ladders (Li-Cor) were employed. Optical density of bands was measured using ImageJ and normalized by the expression of GAPDH or total protein content.

Whole protein extracts from snap-frozen mouse brain tissue were processes as cardiac samples and used as positive control for Western blot analysis involving Na Channel β1-subunit.

### Data Analysis

Data are presented as means ± SE, scatter plots, medians, and interquartile ranges. Fold and percentage changes are referred to median values. Investigators performing acquisition and analysis of data had knowledge of mouse genotype. Statistical analysis was performed using SigmaPlot 11.0 and Graphpad Prism. Data were initially tested for normality (Shapiro–Wilk) and equal variance for assignment to parametric or nonparametric analysis. Parametric tests included Student’s *t* test, paired *t* test, one-way repeated measures analysis of variance (ANOVA) followed by Tukey’s or Holm-Sidak’s multiple comparisons tests. Nonparametric tests included Mann–Whitney rank sum test and Wilcoxon-signed rank test, Friedman repeated-measures analysis of variance on ranks followed by Dunn’s multiple comparisons test (24, 28–30, 41). Fits of regression curves were compared using the extra sum-of-squares *F* test. For categorical data analysis, Fisher’s exact or χ^2^ tests were used. For survival curves, Gehan–Breslow–Wilcoxon and log-rank (Mantel–Cox) tests were employed (32). *P* < 0.05 was considered significant.

## RESULTS

### *Scn1b* Modulates Na^+^ Influx in Adult Ventricular Myocytes

**Figure 1.**
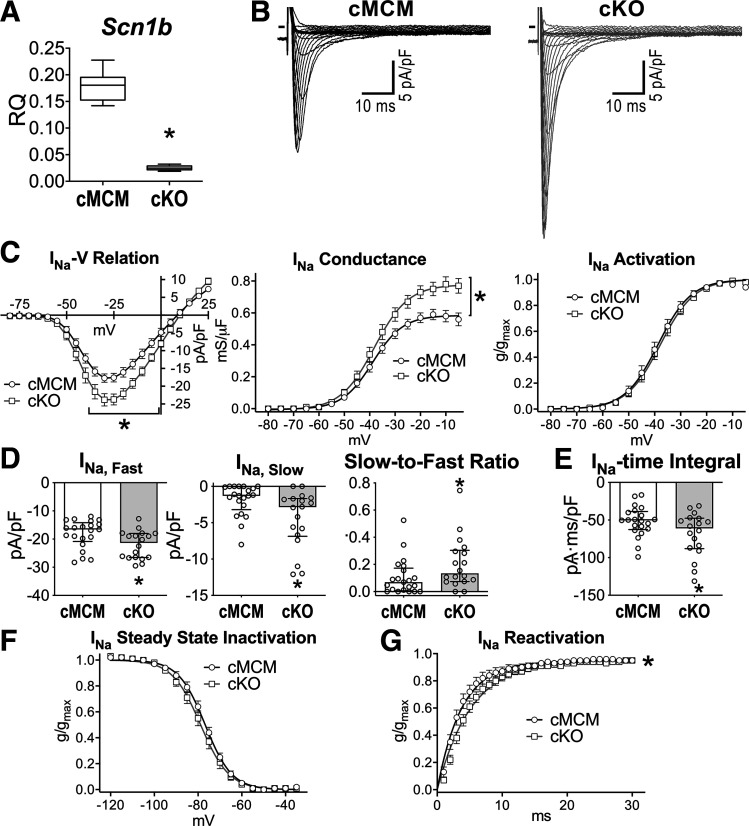
Cardiac specific, adult *Scn1b* deletion enhances Na^+^ influx. *A*: quantitative data for expression of *Scn1b* in cMCM (*n* = 6 cells preparations; 6 male and female mice) and cKO cardiomyocytes (*n* = 6 cells preparations; 6 male and female mice). Data are shown as median and interquartile ranges. *P < 0.001 vs. cMCM, using unpaired *t* test. Animals at more than 4 wk after induction of Cre recombinase expression were employed. *B*: whole cell voltage-gated *I*_Na_ recorded in voltage-clamp in myocytes isolated from cMCM and cKO mouse hearts. The voltage-clamp protocol consisted of a family of depolarizing steps in 5-mV increments. *C*: current-voltage (*I-V*) relations, voltage dependence of conductance, and steady-state activation for *I*_Na_ in myocytes from cMCM (*n* = 21 cells from 5 animals, 2–4 cells/mouse) and cKO mice (*n* = 18 cells from 6 animals, 3–6 cells/mouse). Male and female mice were employed. *P < 0.05 vs. cMCM, using unpaired *t* test. *C* and *D*: quantitative data for properties of *I*_Na_ decay for cells reported in *C*. *P < 0.05 vs. cMCM, using Mann–Whitney test (*D*) or unpaired *t* test (*E*). *F*: steady state inactivation for *I*_Na_ in myocytes from cMCM (*n* = 21 cells from 5 animals, 3–6 cells/mouse) and cKO mice (*n* = 19 cells from 5 animals, 3–5 cells/mouse). *G*: time dependence of reactivation of *I*_Na_ in myocytes from cMCM (*n* = 17 cells from 5 animals, 2–5 cells/mouse) and cKO mice (*n* = 13 cells from 5 animals, 2–3 cells/mouse). Data are shown as means ± SE, median and interquartile ranges with dot plots, or median and interquartile ranges. *P < 0.001 vs. cMCM, using extrasum-of-squares *F* test. Fitting parameters are reported in Supplemental Table S3. cKO, conditional knockout; cMCM, conditional MerCreMer.

To evaluate the role of *Scn1b* gene expression in the adult heart, a rodent model of tamoxifen-inducible MerCreMer recombinase expression (24) leading to *Scn1b* gene deletion in cardiomyocytes (conditional knockout, cKO) was implemented. The cardiac-restricted model and controlled gene expression were used to avoid seizures and lethality of juvenile mice with complete loss of *Scn1b* gene (23) and to minimize the consequences of *Scn1b* deletion in cardiomyocytes during development and early life (4, 9), respectively. Mice with tamoxifen-inducible MerCreMer recombinase expression but lacking the *Scn1b* floxed allele were used as controls (cMCM). After induction of Cre recombinase expression, *Scn1b* transcripts were substantially reduced in cardiomyocytes obtained from cKO hearts, with respect to cells from cMCM animals, whereas transcripts for the voltage-gated Na^+^ channel α-subunits Nav1.5 and Nav1.3 were not affected (Fig. 1*A* and Supplemental Fig. S2*A*). As previously reported (29), levels of proteins for the VGSC β1-subunits were barely detectable in the LV myocardium, by Western blot analysis, preventing the detection of measurable differences between cMCM and cKO (Supplemental Fig. S2*B*).

By whole cell voltage-clamp, Na^+^ current (*I*_Na_) density and channel conductance were increased in cKO myocytes with respect to cMCM cells, whereas steady-state *I*_Na_ activation was comparable for the two groups of cells (Fig. 1, *B* and *C*). By biexponential fitting, the fast (*I*_Na,fast_) and slow (*I*_Na,slow_) components of *I*_Na_ decay were, respectively, 1.3-fold and 2.2-fold larger in cKO myocytes, compared with cMCM cells. This resulted in a larger ratio between the slow and fast components of *I*_Na_ for cKO cells (Fig. 1*D*). Overall, enhancement of *I*_Na,fast_ and *I*_Na,slow_ led to increased Na^+^ influx in cKO cardiomyocytes, evaluated by the time integral of the current during the decay phase (Fig. 1*E*). Steady state inactivation of *I*_Na_ was comparable for the two groups of myocytes but time dependency of recovery from inactivation of *I*_Na_ was slightly delayed by *Scn1b* deletion (Fig. 1, *F* and *G*).

Exposure of cKO myocytes to GS967, an inhibitor of late Na^+^ current (*I*_NaL_) (46, 47), attenuated the amplitude of the *I*_Na_-voltage relation of cKO myocytes (Supplemental Fig. S3*A*). Specifically, the inhibitor reduced *I*_Na,fast_ by 13% and *I*_Na,slow_ by 75%, leading to a 14% reduction of Na^+^ influx in cKO myocytes (Supplemental Fig. S3*B*). Thus, expression of *Scn1b*, which encodes for the Na^+^ channel β1- and β1B-subunits, modulates *I*_Na_ and Na^+^ influx in cardiomyocytes. Consistent with previous results (49), GS967 was found to exert a preferential block on the slow component of *I*_Na_.

### *Scn1b* Modulates the Electrical Recovery of the Entire Heart

**Figure 2.**
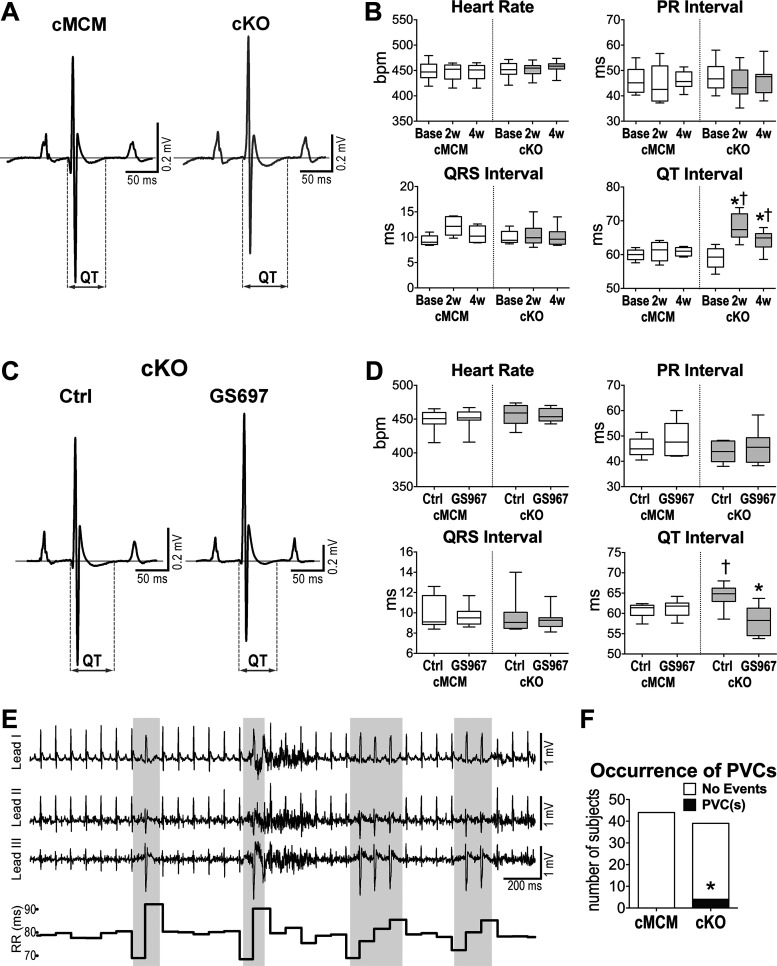
Cardiac specific, adult *Scn1b* deletion prolongs cardiac electrical recovery and affects electrical stability. *A*: electrocardiograms (ECG) obtained in anesthetized male cMCM and cKO mice 4 wk after tamoxifen administration. *B*: quantitative data for electrocardiographic parameters obtained, serially, in cMCM (*n* = 6) and cKO (*n* = 10) male mice before (Base) and 2 wk (2w) and 4 wk (4w) after tamoxifen treatment. Data are shown as median and interquartile ranges. *P < 0.01 vs. Base for the same genotype, using one-way repeated measures ANOVA followed by Tukey’s multiple comparison test. †*P* < 0.05 vs. cMCM at the same time point, using unpaired *t* test. *C*: ECGs obtained in anesthetized male cKO mouse 4 wk after gene deletion, before (control, Ctrl, *left* trace) and after administration of the *I*_NaL_ inhibitor GS967 (0.5 mg/kg body wt ip, *right* trace). *D*: quantitative data for electrocardiographic parameters obtained in male cMCM (*n* = 9) and cKO (*n* = 8) mice 4 wk after gene deletion before (Ctrl) and after administration of GS967. Data are shown as median and interquartile ranges. *P < 0.01 vs. Ctrl, using Wilcoxon test. †*P* < 0.01 vs. cMCM in the same experimental condition, using Mann–Whitney test. *E*: ECG traces (lead I, II, and III) and R-R interval duration collected in a conscious cKO mouse. Gray areas identify ventricular ectopic beats. *F*: quantitative data for occurrence of arrhythmic events in cMCM (*n* = 44; 29 males, 15 females) and cKO (*n* = 39; 26 males, 13 females) mice 4–8 wk after tamoxifen treatment. *P < 0.05 vs. MCM with both Fisher’s exact test and χ^2^ test. cKO, conditional knockout; cMCM, conditional MerCreMer, MCM, MerCreMer.

To establish the consequences of *Scn1b* deletion and enhanced Na^+^ influx in cardiomyocytes on the in vivo electrical properties of the adult heart, electrocardiograms (ECGs) were serially obtained in the anesthetized state in a cohort of cMCM and cKO male mice before (baseline) and at 2 and 4 wk after induction of genomic recombination. Atrioventricular conduction (PR interval) and ventricular activation (QRS duration) were similar for the two groups of mice at baseline and were not altered following genomic recombination. In contrast, electrical repolarization, assessed by the QT interval duration, was comparable at baseline for the two genotypes, but became prolonged at 2 (14% increase) and 4 (10% increase) wk in male cKO mice, but not in male cMCM animals (Fig. 2, *A* and *B*). Similar findings were observed in a cohort of female mice following *Scn1b* gene deletion (Supplemental Fig. S4*A*).

To establish the contribution of Na^+^ influx and specifically *I*_NaL_ on the protracted electrical recovery of cKO mice, ECGs were collected in cKO and cMCM animals before (control, Ctrl) and after administration of GS967 (32). For both male and female animals for the two genotypes, the *I*_NaL_ inhibitor had no consequences on atrioventricular conduction and ventricular activation, but instead shortened (by 10%–14%) and normalized the QT interval in cKO mice (Fig. 2, *C* and *D* and Supplemental Fig. S4*B*). The inhibitor had no effects on the electrical repolarization of cMCM animals. In addition, mexiletine, another Na^+^ channel blocker that preferentially inhibits *I*_NaL_ (29), was equally effective in normalizing electrical repolarization in cKO male animals (Supplemental Fig. S5).

To define whether the delayed repolarization of cKO hearts affected electrical stability, ECGs were collected in the conscious state in male and female mice for the two genotypes, for a period of 10 min (32). Ectopic events did not occur in cMCM mice (*n* = 44), whereas single or multiple premature ventricular complexes (PVCs) were observed in 4 of the 39 (10.3%) cKO mice examined (Fig. 2, *E* and *F*). However, tests designed to evaluate the arrhythmogenicity of ex vivo, perfused organs from male and female animals, revealed no differences in the inducibility of ectopic events for cKO or cMCM hearts, by using programmed electrical stimulation (Supplemental Fig. S6). Thus, loss of *Scn1b* results in slower ventricular repolarization, which is mediated, at least in part, by enhanced *I*_NaL_, but has no major consequences of the propensity of the adult heart to develop arrhythmic events.

### *Scn1b* Modulates Diastolic Function

**Figure 3.**
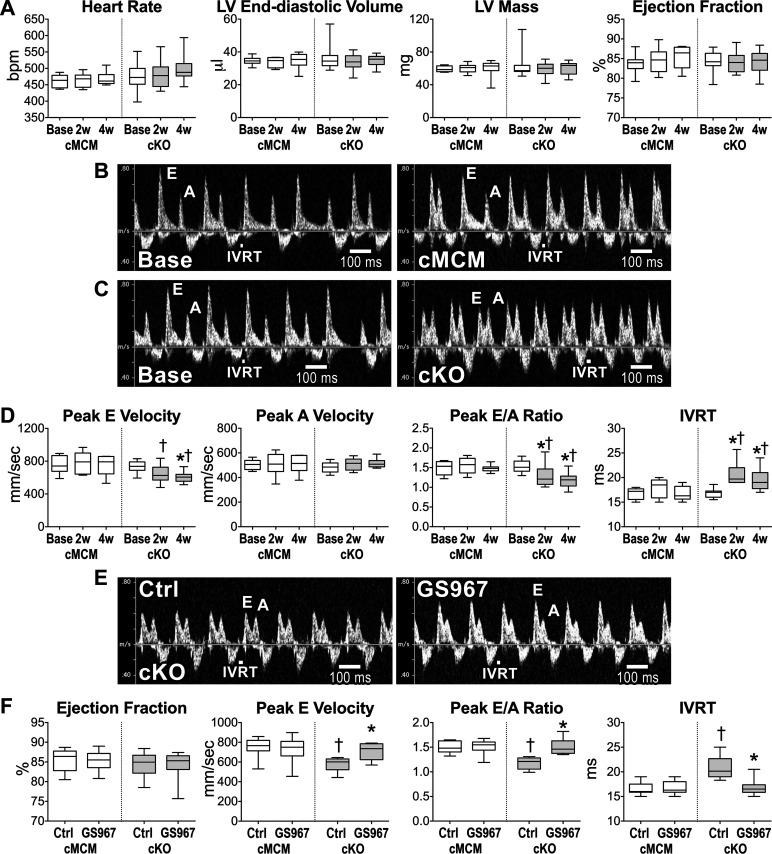
Cardiac specific, adult *Scn1b* deletion alters diastolic function. *A*: quantitative data for two-dimensional echocardiographic parameters obtained in cMCM (*n* = 8) and cKO (*n* = 13) male mice before (Base) and 2 wk (2w) and 4 wk (4w) after tamoxifen treatment. Data are shown as median and interquartile ranges. *B*: transmitral flow Doppler echocardiograms for a male cMCM mouse before (Base, *left*) and 4 wk (*right*) after tamoxifen treatment. *C*: transmitral flow Doppler echocardiograms for a male cKO mouse before (Base, *left*) and 4 wk (*right*) after *Scn1b* deletion. *D*: quantitative data for transmitral flow Doppler echocardiographic parameters obtained in cMCM and cKO male mice shown in *A* are reported as median and interquartile ranges. *P < 0.01 vs. Base for the same genotype, using one-way repeated measures ANOVA followed by Tukey’s multiple comparison test. †*P* < 0.05 vs. cMCM at the same time point, using unpaired *t* test. *E*: transmitral flow Doppler echocardiograms for a male cKO mouse at 4 wk after gene deletion, before (Ctrl, *left*) and after administration of the *I*_NaL_ inhibitor GS967 (0.5 mg/kg body wt ip, *right*). *F*: quantitative data for two-dimensional and transmitral flow Doppler echocardiographic parameters obtained in male cMCM (*n* = 13) and cKO (*n* = 8) mice 4–6 wk after gene deletion before (Ctrl) and after administration of GS967. Data are shown as median and interquartile ranges. *P < 0.01 vs. Ctrl, using paired *t* test. †*P* < 0.001 vs. cMCM in the same experimental condition, using unpaired *t* test. A, active filling wave; cKO, conditional knockout; cMCM, conditional MerCreMer; *E*, early passive filling wave; IVRT, isovolumic relaxation time.

To establish the consequences of *Scn1b* deletion and increased Na^+^ influx in cardiomyocytes on the systolic and diastolic aspects of cardiac function, two-dimensional and Doppler echocardiography were serially performed before (baseline) and after induction of genomic recombination in a cohort of cMCM and cKO mice. For both male and female animals, left ventricular (LV) end diastolic volume, LV mass, and ejection fraction (EF) were preserved from baseline to 4 wk after tamoxifen administration (Fig. 3*A* and Supplemental Fig. S7*A*). By transmitral flow Doppler, the peak velocity of the early passive (E wave) and active (A wave) LV filling, E/A ratio, and isovolumic relaxation time (IVRT) were evaluated as diastolic indices. For the two sexes, these parameters were not altered in cMCM mice at 2 and 4 wk following gene recombination, whereas cKO mice experienced a substantial attenuation of the passive filling (E wave, 15%–24% reduction) and *E/A* ratio (20%–28% reduction), together with prolongation (12%–31% increase) of IVRT (Fig. 3, *B–D* and Supplemental Fig. S7*B*). At 4 wk after *Scn1b* deletion, inhibition of *I*_NaL_ with GS967 in both male and female mice or with mexiletine in male animals reversed defective indices of diastolic function, without interfering with ejection fraction (Fig. 3, *E* and *F* and Supplemental Fig. S7, *C* and *D*). No effects of *I*_NaL_ inhibition were observed in cMCM male or female mice.

**Figure 4.**
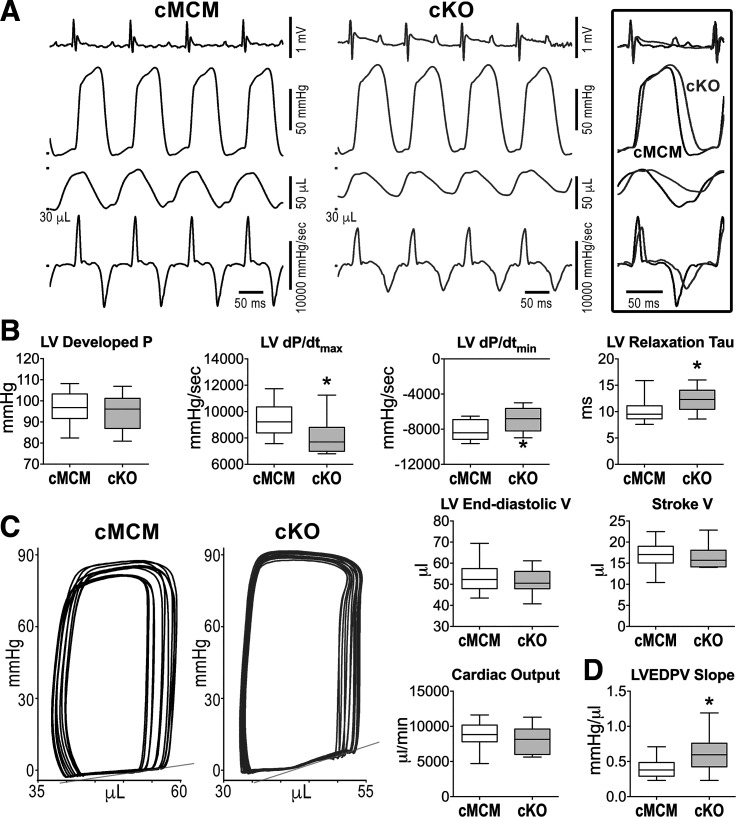
Cardiac specific, adult *Scn1b* deletion alters rate of pressure development and decay and LV compliance. *A*: traces of ECG, LV pressure, LV volume, and first derivative of LV pressure (dP/d*t*) for male cMCM and cKO mice. Traces are superimposed in the inbox. *B*: quantitative data for LV hemodynamic and volumetric parameters obtained in cMCM (*n* = 17) and cKO (*n* = 13) male mice are shown as median and interquartile ranges. *P < 0.05 vs. cMCM, using unpaired *t* test or Mann–Whitney test. *C*: PV loops obtained during occlusion of the inferior vena cava to unload the LV in a male cMCM mouse and a male cKO mouse. Gray lines reflect the slope of the LV end diastolic PV (LVEDPV) relationship. *D*: Quantitative data for LVEDPV slope obtained in cMCM (*n* = 13) and cKO (*n* = 12) male mice are shown as median and interquartile ranges. *P < 0.05 vs. cMCM, using unpaired *t* test. cKO, conditional knockout; cMCM, conditional MerCreMer; ECG, electrocardiogram; LV, left ventricle.

To further evaluate the functional effects of *Scn1b* deletion on cardiac function, LV hemodynamics and pressure-volume (PV) loops were assessed in the closed chest preparation in male mice after gene recombination. Developed pressure was comparable between cKO and cMCM male mice, but maximal rates of pressure raise and decay, indicative of the velocity of ventricular contraction and relaxation, were both reduced in cKO mice by 17%–19% (Fig. 4, *A* and *B*). Similarly, the time constant of pressure decay (tau) was increased by 30% in cKO animals. By PV assessment, LV end-diastolic volume, stroke volume, and cardiac output were similar in the two groups of animals. However, slope of the LV end-diastolic PV curve, obtained following unloading of the LV ventricle (29), was 1.6-fold steeper in cKO mice (Fig. 4, *C* and*D*), supporting the notion that *Scn1b* modulates ventricular compliance. Similar results were obtained in female mice for the two genotypes (Supplemental Fig. S8).

At euthanasia, LV weight and heart weight-to-body weight ratio were similar in cMCM and cKO mice, for the two sexes (Supplemental Fig. S9, *A* and *B*). Similarly, myocyte size, assessed in isolated cell preparations, and levels of interstitial fibrosis in the LV myocardium were comparable in cMCM and cKO male mice (Supplemental Fig. S9, *C*–*E*). These results indicate that perturbations of LV filling and ventricular stiffness in cKO mice are not associated with anatomical or structural changes of the myocardium. Thus, loss of *Scn1b* interferes with diastolic function, a process that is mediated, at least in part, by enhanced Na^+^ influx in cardiomyocytes.

### Enhanced Na^+^ Influx in Cardiomyocytes Alters Cardiac Function Ex Vivo

To clarify the effects of enhanced Na^+^ influx on diastolic function and ventricular compliance, which is relevant for mice with *Scn1b* deletion, hearts from C57Bl/6N male mice at ∼3 mo of age were studied in an ex vivo system, using Langendorff perfusion and LV balloon technique (30). Anemonia toxin II (ATX-II, 5 nmol/L) was employed to enhance the slow-inactivating *I*_NaL_ in myocytes (15, 29, 41) while evaluating isometric LV pressure and electrical activity of the heart. In organs paced at 8 Hz, exposure to ATX-II led to a progressive increase in isovolumic systolic and diastolic LV pressure, effects largely reversible upon washout (Supplemental Fig. S10*A*). Quantitatively, there was a 1.4-fold increase in systolic and developed pressure, together with a threefold elevation in diastolic pressure (Supplemental Fig. S10*B*). To better define the influence of *I*_NaL_ on the passive, diastolic properties of the myocardium, LV volume was progressively increased in paced hearts and diastolic pressure-volume (PV) relationships were evaluated before and after perfusion with ATX-II. With respect to baseline conditions, enhancement of *I*_NaL_ shifted the diastolic PV curve to higher passive pressure levels (Supplemental Fig. S10*C*). Thus, under isovolumic experimental conditions, the acute increase in Na^+^ influx in cardiomyocytes potentiates LV systolic function but largely interferes with diastolic compliance of the ventricular myocardium.

### *Scn1b* Modulates Intracellular Ca^2+^ Cycling and Kinetics of Cell Shortening and Relengthening

**Figure 5.**
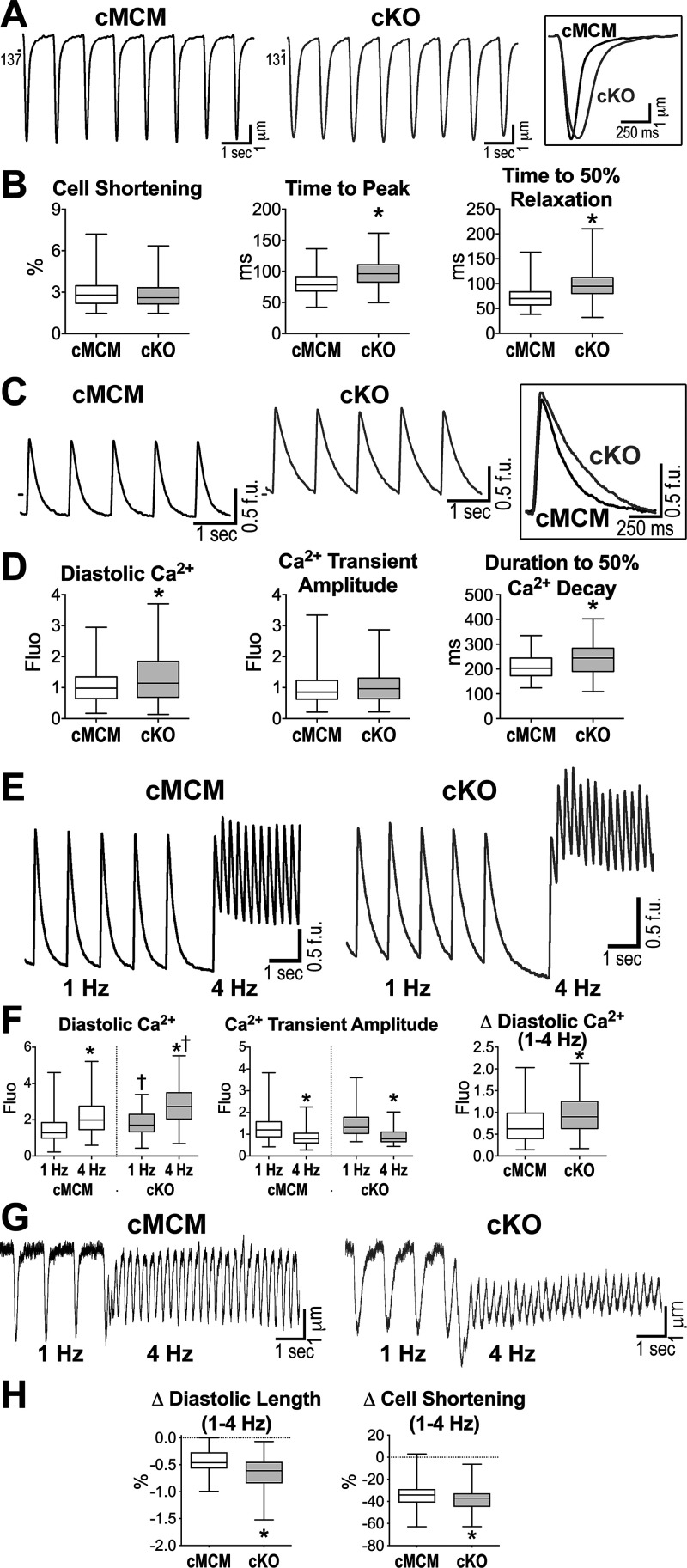
Cardiac specific, adult *Scn1b* deletion alters cell mechanics and Ca^2+^ homeostasis in myocytes. *A*: cell shortening traces from myocytes obtained from male cMCM and cKO mice. Superimposed traces are reported in the inset. *B*: quantitative data for cell shortening in cMCM (*n* = 283 cells, 5 animals, 44–60 cells/mouse) and cKO myocytes (*n* = 372 cells, 7 animals 40–59 cells/mouse) obtained from male mice are shown as median and interquartile ranges. *P < 0.0001 vs. cMCM, using unpaired *t* test or Mann–Whitney test. *C*: traces of Ca^2+^ transients from myocytes obtained from cMCM and cKO male mice. Superimposed traces are reported in the inset. *D*: quantitative data for Ca^2+^ transients in cMCM (*n* = 284 cells, 5 animals, 52–60 cells/mouse) and cKO myocytes (*n* = 332 cells, 6 animals, 44–60 cells/mouse) obtained from male mice are shown as median and interquartile ranges. *P < 0.01 vs. cMCM, using Mann–Whitney test. *E*: traces of Ca^2+^ transients from cMCM and cKO myocytes stimulated at 1 Hz and 4 Hz. *F*: quantitative data for Ca^2+^ transients at 1 Hz and 4 Hz in cMCM (*n* = 120 cells, 5 animals, 17–34 cells/mouse) and cKO (*n* = 76 cells, 5 animals, 8–34 cells/mouse) myocytes obtained from male and female mice are shown as median and interquartile ranges. *P < 0.0001 vs. 1 Hz, using Wilcoxon test or paired *t* test; †*P* < 0.0001 vs. cMCM at the same stimulation frequency, using Mann–Whitney test. In graph Δ Diastolic Ca^2+^, *P < 0.0001 vs. cMCM, using Mann–Whitney test. *G*: traces of cell shortening from cMCM and cKO myocytes stimulated at 1 Hz and 4 Hz. *H*: quantitative data for variation (Δ) of diastolic cell length and cell shortening at 4 Hz with respect to 1 Hz in cMCM (*n* = 56 cells, 5 animals, 9–18 cells/mouse) and cKO (*n* = 72 cells, 5 animals, 8–20 cells/mouse) myocytes are shown as median and interquartile ranges. Male and female mice were used. *P < 0.05 vs. cMCM, using unpaired *t* test. cKO, conditional knockout; cMCM, conditional MerCreMer; f.u., fluorescent unit.

The findings that *I*_NaL_ inhibition normalized electrical repolarization and corrected diastolic function in cKO mice suggested that defective myocyte mechanics, secondary to the enhancement of Na^+^ influx, represents the basis for the impaired relaxation of the LV in mice following the loss of *Scn1b*. To test this possibility, cell shortening and intracellular Ca^2+^ levels were evaluated in isolated myocyte preparations obtained from cMCM and cKO male mice at more than 4 wk after gene recombination. Under field stimulation, percent cell shortening was comparable in the two groups of cells, but cKO myocytes presented delayed time to peak shortening (22% increase) and protracted relaxation (35% increase of time to 50% relaxation), with respect to cMCM cells (Fig. 5, *A* and *B*). Similarly, kinetics of the Ca^2+^ transient, quantified as time of the Ca^2+^ transient to reach 50% of its decay, were protracted (20% increase) in cKO myocytes, with respect to cMCM cells. Interestingly, the Ca^2+^ transient amplitude was similar between genotypes, but diastolic Ca^2+^ levels, evaluated by the fluorescent signal of the Ca^2+^ indicator, were higher (16% increase) in cKO myocytes (Fig. 5, *C* and *D*). Similar findings were obtained in myocytes from cMCM and cKO female mice (Supplemental Fig. S11).

To test the possibility that defective cytosolic Ca^2+^ clearance is involved in defective intracellular Ca^2+^ homeostasis and cell mechanics of cKO myocytes, Ca^2+^ levels were evaluated, acutely, in response to an increase in the stimulation rate from 1 Hz to 4 Hz. Based on similarities for the two sexes, myocytes obtained from male and female mice were combined. This protocol allowed us to assess the efficiency of the clearance process in the two cell types. At both stimulation frequencies, the Ca^2+^ transient amplitude was similar between genotypes, but diastolic Ca^2+^ levels were higher in cKO myocytes (Fig. 5, *E* and *F*). Importantly, with respect to cMCM cells, the increase in diastolic Ca^2+^ from 1 to 4 Hz was 43% larger in cKO cells, strengthening the notion that loss of *Scn1b* interferes with clearance of cytosolic Ca^2+^ in cKO myocytes.

Cell contractility tests, performed with male and female myocytes, revealed that the increase in stimulation rate from 1 to 4 Hz was coupled with a greater extent of cell contracture for cKO myocytes (33% more contracted), measured as variation of diastolic length, and weaker (8% reduction) shortening (Fig. 5, *G* and *H*). At 30 s of stimulation at 4 Hz, however, cell shortening was comparable in the two groups of myocytes, but contraction and relaxation kinetics were delayed (prolonged by 7 and 14%, respectively) in cKO cells (Supplemental Fig. S12).

**Figure 6.**
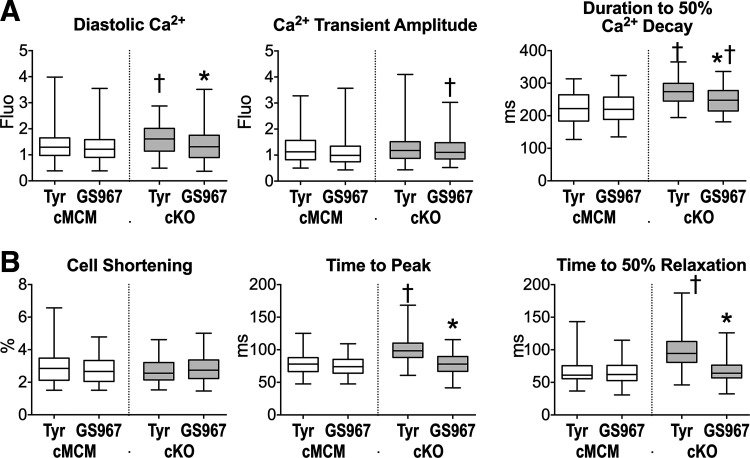
Inhibition of *I*_NaL_ ameliorates kinetics of Ca^2+^ cycling and cell mechanics in myocytes with *Scn1b* deletion. *A*: quantitative data for Ca^2+^ transients in cMCM and cKO myocytes in Tyrode solution alone (Tyr, *n* = 102, 119, respectively) or with 300 nmol/L GS967 (*n* = 105, 123, respectively) are shown as median and interquartile ranges. Cells were obtained from male and female cMCM (*n* = 5, 8–36 cells/mouse) cKO (*n* = 5, 15–47 cells/mouse) mice. *P < 0.01 vs. Tyr, using unpaired *t* test or Mann–Whitney test; †*P* < 0.01 vs. cMCM in the same experimental condition, using Mann–Whitney test. *B*: quantitative data for cell mechanics in cMCM and cKO myocytes in Tyrode solution alone (Tyr, *n* = 76, 157, respectively) or with 300 nmol/L GS967 (*n* = 79, 128, respectively) are shown as median and interquartile ranges. Cells were obtained from male and female cMCM (*n* = 4, 13–20 cells/mouse) cKO (*n* = 4, 10–46 cells/mouse) mice. *P < 0.0001 vs. Tyr, using Mann–Whitney test; †*P* < 0.0001 vs. cMCM in the same experimental condition, using Mann–Whitney test. cKO, conditional knockout; cMCM, conditional MerCreMer.

To clarify the role of *I*_NaL_ in the altered intracellular Ca^2+^ homeostasis of cKO myocytes, cells were exposed to GS967. For the two genotypes, cells obtained from male and female mice were combined. The *I*_NaL_ inhibitor had no major effects on cMCM myocytes but reduced by 18% diastolic Ca^2+^ levels and accelerated by 10% kinetics of Ca^2+^ transients in cKO myocytes, attenuating differences observed between the two groups of cells (Fig. 6*A*). Similarly, GS967 had no detectable effects on cell mechanics in cMCM myocytes, but it accelerated dynamics of cell shortening (by 21%) and relaxation (by 32%) in cKO myocytes, normalizing their contractile behavior (Fig. 6*B*). Comparable effects on Ca^2+^ cycling and cell shortening of cMCM and cKO myocytes from male and female mice were observed with the *I*_NaL_ inhibitor mexiletine (Supplemental Fig. S13). Therefore, *Scn1b* gene expression modulates intracellular Ca^2+^ homeostasis and kinetics of myocyte contraction and relaxation, partly by affecting sodium influx.

### *Scn1b* Modulates Electrical Repolarization

**Figure 7.**
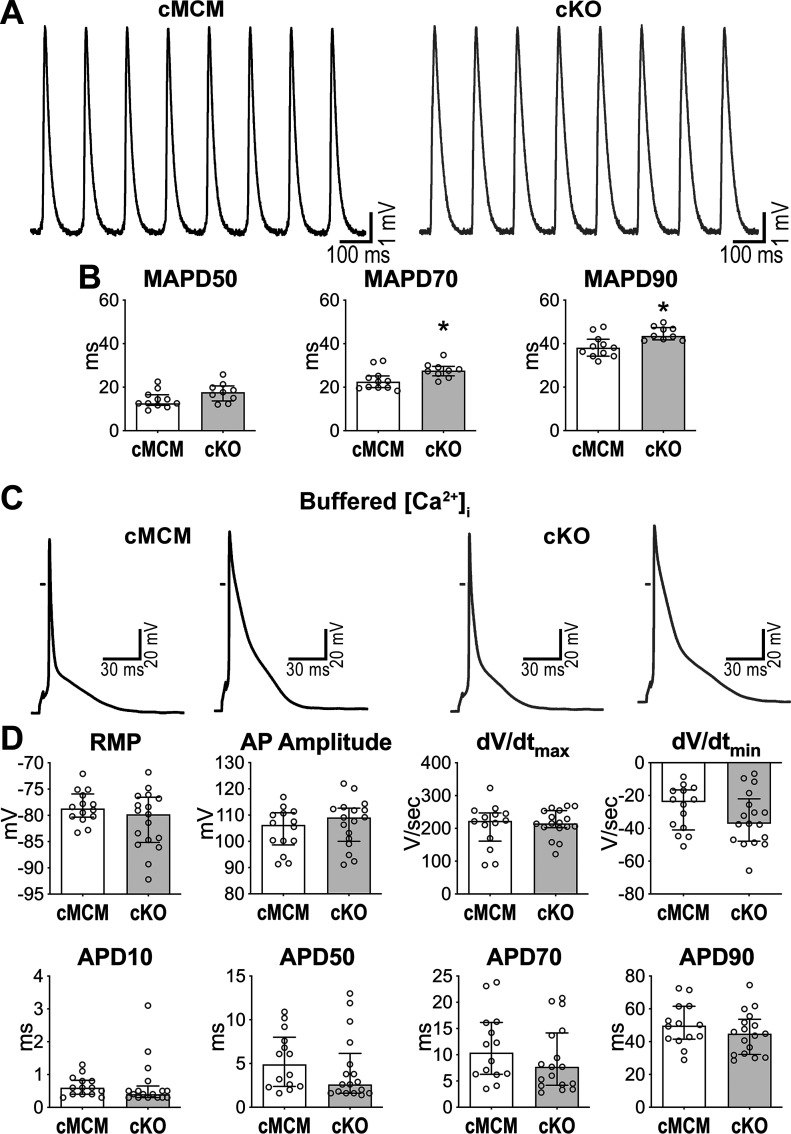
Cardiac specific, adult *Scn1b* deletion prolongs myocardial repolarization. *A*: monophasic APs obtained from the LV myocardium of perused hearts from cMCM and cKO male mice stimulated at 8 Hz. *B*: quantitative data for time to 50 (MAP50), 70 (MAP70), and 90% (MAP90) repolarization of the MAP obtained in the LV myocardium of cMCM (*n* = 11) and cKO (*n* = 9) male hearts are shown as median and interquartile ranges with dot plots. *P < 0.05 vs. cMCM, using unpaired *t* test. *C*: APs elicited at 1 Hz in the presence of buffered [Ca^2+^]_i_ in myocytes obtained from the LV of cMCM and cKO male hearts. *D*: quantitative data for AP properties elicited at 1 Hz pacing rate in the presence of buffered [Ca^2+^]_i_. Data obtained from LV myocytes from cMCM (*n* = 14 cells from 4 animals, 2–5 cells/mouse) and cKO (*n* = 17 cells from 4 animals, 3–6 cells/mouse) male mice are shown as median and interquartile ranges with dot plots. AP, action potential; cKO, conditional knockout; cMCM, conditional MerCreMer; LV, left ventricle; MAP, monophasic action potential.

To establish whether the delayed cardiac electrical recovery of cKO mice was mediated by prolonged local repolarization of the myocardium, monophasic action potentials (MAPs) were collected on the epicardial aspect of the LV free wall of perfused hearts, obtained from cMCM and cKO mice. At 8-Hz pacing rate, the initial portion of the repolarization phase of the MAP was comparable in cMCM and cKO tissue, whereas time to 70 and 90% of the repolarization phase were ∼14%–23% longer in cKO male and female hearts, with respect to organs from sex-matched cMCM animals (Fig. 7, *A* and *B* and Supplemental Fig. S14). To evaluate whether the altered intracellular Ca^2+^ homeostasis of cKO myocytes played a role on the late repolarization phase of the AP, current-clamp measurement was performed in isolated male cardiomyocytes under [Ca^2+^]_i_-buffered conditions designed to prevent changes in cytosolic Ca^2+^ (39, 45). Under this experimental situation, ranges of AP duration were comparable in cMCM and cKO myocytes (Fig. 7, *C* and *D*), abrogating repolarization differences observed locally in the myocardium and entire heart. Thus, *Scn1b* gene expression appears to modulate the repolarization of the myocardium by interfering with intracellular ionic homeostasis.

### *Scn1b* and Ca^2+^ Handling Machinery

**Figure 8.**
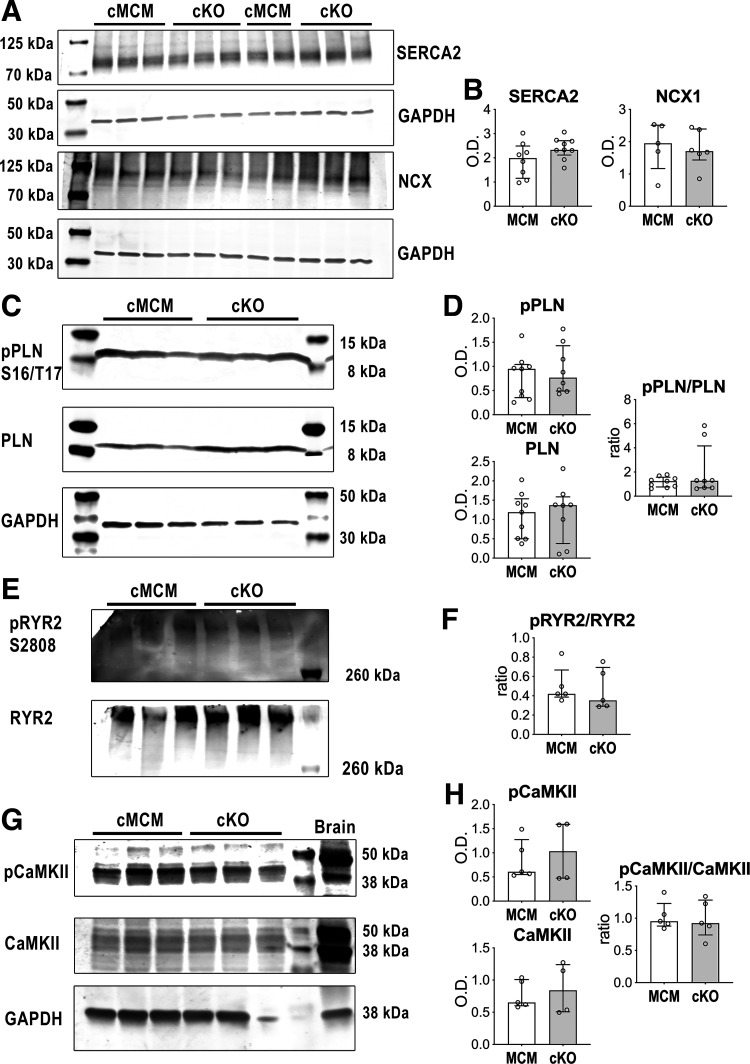
Cardiac specific, adult *Scn1b* deletion and screening of Ca^2+^ handling proteins. *A*: expression of SERCA2a and NCX1 the LV myocardium of cMCM and cKO male mice, by Western blotting. GAPDH is the loading condition. *B*: quantitative data for expression of SERCA2a and NCX1 in the LV myocardium of cMCM (*n* = 8, 5) and cKO (*n* = 9, 6) male mice are reported as median and interquartile ranges with dot plots. *C*: expression of phosphorylated (serine 16/threonine 17, pPLN S16/T17) and total phospholamban (PLN) proteins in the LV myocardium of cMCM and cKO male mice, by Western blotting. GAPDH is the loading condition. *D*: quantitative data for expression of phosphorylated (pPLN) and total (PLN) phospholamban, and pPLN-to-PLN ratio in the LV myocardium of cMCM (*n* = 9) and cKO (*n* = 8) male mice are reported as median and interquartile ranges with dot plots. *E*: expression of phosphorylated (serine 2808, pRYR2 S2808) and total ryanodine (RYR2) proteins in the LV myocardium of cMCM and cKO male mice, by Western blotting. *F*: quantitative data for pRYR2-to-RYR2 ratio in the LV myocardium of cMCM (*n* = 5) and cKO (*n* = 5) male mice are reported as median and interquartile ranges with dot plots. *G*: expression of phosphorylated (threonine 286, pCaMKII) and total CaMKII proteins in the LV myocardium of cMCM and cKO male mice, by Western blotting. GAPDH is the loading condition. *H*: quantitative data for expression of phosphorylated (pCaMKII) and total CaMKII, and pCaMKII-to-CaMKII ratio in the LV myocardium of cMCM (*n* = 5) and cKO (*n* = 4–5) male mice are reported as median and interquartile ranges with dot plots. CaMKII, Ca^2+^-calmodulin-dependent protein kinase II; cKO, conditional knockout; cMCM, conditional MerCreMer; LV, left ventricle; O.D., optical density; RYR2, ryanodine receptor; SERCA2a, sarco(endo)plasmic reticulum calcium ATP-ase 2.

To evaluate the possibility that alterations of Ca^2+^ handling proteins contributed to the delayed kinetics of Ca^2+^ transients and cell contraction and relaxation in myocytes with *Scn1b* deletion, an initial screening of key proteins involved in intracellular Ca^2+^ homeostasis was conducted by Western blot analysis, in LV myocardium from cMCM and cKO mice. Levels of the sarco(endo)plasmic reticulum calcium ATP-ase 2 (SERCA2) and Na^+^/Ca^2+^ exchanger (NCX), which are involved, respectively, in Ca^2+^ reuptake into the SR and cytosolic Ca^2+^ extrusion, were comparable in the two groups (Fig. 8, *A* and *B*). Similar level of transcripts for SERCA2 and NCX were also found in LV myocytes from cMCM and cKO animals (Supplemental Fig. S15). Moreover, phosphorylation status of phospholamban (PLN) and ryanodine receptor (RYR2), molecules involved in reuptake and release of Ca^2+^ into and from the sarcoplasmic reticulum (SR), respectively, were assessed. Specifically, by considering phosphorylation sites target of both protein kinase A and the Ca^2+^-calmodulin-dependent protein kinase II (CaMKII) (50, 51), no differences were observed between cMCM and cKO tissue for phosphorylated-to-total protein ratio for PLN and for RYR2 (Fig. 8, *C–F*). Moreover, expression and phosphorylation levels of CaMKII, which is involved in regulating Ca^2+^ handling molecules, were comparable in the myocardium from the two groups of mice (Fig. 8, *G* and *H*).

Overall, this analysis at the molecular level suggests that *Scn1b* gene deletion does not affect Ca^2+^ handling proteins.

## DISCUSSION

Results of the current study document that *Scn1b*, encoding the β1- and β1B-subunits of the VGSC, is an important regulator of myocyte and cardiac function in adult life. Expression of *Scn1b* controls magnitude and modality of Na^+^ influx in cardiomyocytes with repercussions on intracellular Ca^2+^ homeostasis and cell mechanics. The modulatory actions of *Scn1b* at the cellular level are accompanied, in vivo, by effects on ventricular repolarization and diastolic stiffness. Mechanistically, reported downstream effects of *Scn1b* gene expression appear to be secondary, at least in part, to the consequences of Na^+^ entry on intracellular ionic balance.

Myocytes from cKO hearts have enhanced Na^+^ influx, mediated by increases of both fast- and slow-inactivating components of *I*_Na_, mirroring results previously obtained in systemic *Scn1b*-null neonatal mice (9) and adult animals with constitutively active cardiac-restricted *Scn1b* deletion (8). With respect to control cells, cKO myocytes present slower Ca^2+^ transient decay and elevated diastolic Ca^2+^ levels, together with delayed kinetics of cell contraction and relaxation. These defective properties are consistent, at least in part, with consequences of increased Na^+^ entry on cytoplasmic Ca^2+^ load, an effect mediated by the altered modality of operation of the Na^+^/Ca^2+^ exchanger (NCX), occurring in the presence of elevated intracellular Na^+^ concentration ([Na^+^]_i_) (13–15). In fact, enhanced [Na^+^]_i_ reduces the chemical gradient of this cation across the membrane, and shifts the reversal potential for operation of NCX to more negative values (52, 53). Thus, enhanced [Na^+^]_i_ favors Ca^2+^ entry via NCX reverse mode and attenuates Ca^2+^ extrusion via NCX forward mode, interfering with cytoplasmic Ca^2+^ clearance. The possibility that this scenario is operative in myocytes lacking *Scn1b* is corroborated by results with *I*_NaL_ inhibitors GS967 and mexiletine, in which pharmacological reduction of Na^+^ entry normalized Ca^2+^ cycling and restored contractile kinetics of cKO myocytes. Importantly, inhibition of *I*_NaL_ was equally effective in reversing repolarization and diastolic defects observed in cKO mice. At the molecular level, no alterations were observed for ryanodine receptors, mediating Ca^2+^ release from the SR to the cytoplasm, SERCA and phospholamban, involved in Ca^2+^ reuptake into the SR, and NCX, responsible for cytosolic Ca^2+^ extrusion during diastole. These results reinforce the possibility that changes in intracellular Na^+^ homeostasis contribute to the Ca^2+^ cycling imbalance observed in cKO myocytes. Overall, results collected at the cellular level support the notion that enhanced Na^+^ influx is a key event associated with the in vivo manifestation of loss of *Scn1b* gene expression.

Diastolic function and pattern of LV filling largely depend on myocardial passive tension, a property that is dictated by the level of muscle hypertrophy, collagen deposition, and/or intrinsic myocyte stiffness (54, 55). The latter depends, at least in part, on posttranslational modifications and isoform abundance of the cytoskeletal protein titin (55). Moreover, defective myocardial relaxation arising from *1*) altered kinetics of Ca^2+^ dissociation from troponin C, *2*) reduced rate of myofilament cross bridge detachment, and/or *3*) ineffective cytosolic Ca^2+^ clearance provides residual active tension to the muscle during diastole, interfering with chamber distensibility and proper LV filling (54, 55). In this regard, the possibility that enhanced Na^+^ influx and perturbation of intracellular Ca^2+^ homeostasis contribute to the manifestation of diastolic dysfunction has emerged in recent years (14, 56). Results of this study using cardiac specific *Scn1b*-null mice and ex vivo hearts with enhanced *I*_NaL_ support the causative link between increased Na^+^ influx and defective diastolic function.

Impaired diastole in cardiac specific *Scn1b*-null mice was documented by perturbed LV filling pattern and increased isovolumic relaxation time, by Doppler echocardiography, together with attenuated rate and time constant of LV pressure decay, and steep slope of the LV end-diastolic pressure-volume relation, assessed by cardiac catheterization. These defects occurred in the absence of alterations in chamber volume, LV mass, interstitial fibrosis, and myocyte volume, weakening the possibility that structural modifications of the myocardium contributed to the appearance of impaired diastolic function. Importantly, diastolic indices were ameliorated following inhibition of Na^+^ influx in cardiac specific *Scn1b*-null mice, suggesting that defective myocardial relaxation secondary to dysregulated Na^+^ homeostasis represents a dynamic component to LV stiffness that can be targeted therapeutically. Clinically, inhibition of *I*_NaL_ in patients with diastolic dysfunction has provided promising results (20–22), but a recent trial has reported no overall effects of this intervention in patients with nonobstructive hypertrophic cardiomyopathy (57). These clinical results question the efficacy of *I*_NaL_ inhibitors to ameliorate the functional capacity of the hypertrophic heart. However, the understanding of the exact contribution of Na^+^ influx on the manifestation of diseased conditions that are multifactorial in nature may provide better rationale for the use of *I*_NaL_ blockers in the patient population with diastolic dysfunction.

Heart failure is a progressive disease often involving asymptomatic diastolic defects before the manifestation of overt organ failure (58, 59). But whether impaired LV filling constitutes an aggravating factor or the central event in the evolution of cardiac decompensation remains to be established. Interestingly, diastolic defects in cardiac specific *Scn1b*-null mice occurred without alterations in ejection fraction, cardiac output, or LV mass, possibly reflecting asymptomatic conditions found in humans. Moreover, young-adult male and female mice were similarly affected by the loss of *Scn1b* in the heart, a condition that may change after reaching reproductive senescence, which, in mice, occurs at 9–12 mo of age (60). Thus, experiments involving the imposition of risk factors on cardiac specific *Scn1b*-null animals, including age and female sex, are granted and may clarify the consequences of enhanced Na^+^ influx and diastolic impairment on the development of heart failure.

*SCN1B* gene variants in humans have been implicated in inherited neurological disorders and cardiac arrhythmias, including Brugada, long QT, and sudden infant death syndromes (61). Although no clinical information is available on the consequences of these rare genetic diseases on ventricular performance, it has been reported that patients with long QT syndrome type 3, an inherited condition due to *SCN5A* gene variants and enhanced *I*_NaL_, present diastolic dysfunction assessed by echocardiography (62). Thus, our experimental observations in mice with enhanced Na^+^ influx provide mechanistic insights to human pathologies involving gain-of-function variants or modifications of Na^+^ channels.

Consistent with finding by Lopez-Santiago (9) and Lin (8) using constitutively active models, loss of *Scn1b* gene in adult life prolonged QT intervals of the in vivo ECG and protracted duration of epicardial monophasic action potentials of the ex vivo perfused hearts, with respect to control animals. Inhibition of *I*_NaL_ normalized QT interval duration in cKO mice, pointing to excessive Na^+^ influx as a contributing factor for the protracted repolarization associated with cardiac loss of the *Scn1b* gene. Interestingly, no major differences were observed in the duration of the action potential for cMCM and cKO myocytes analyzed under [Ca^2+^]_i_-buffered conditions. This intervention, designed to prevent changes in cytosolic Ca^2+^ (39, 45) and attenuating NCX function (63), suggests that dysregulated intracellular Ca^2+^ homeostasis is involved in repolarization defects coupled with loss of *Scn1b*.

Lin et al. (8) reported that ex vivo hearts from constitutively active *Scn1b* null mice have increased susceptibility to polymorphic ventricular arrhythmias, whereas hearts from cKO and cMCM mice studied here presented comparable propensity to develop arrhythmic events, using programmed electrical stimulation. However, we found that, in conscious animals under restrained condition, ∼10% of cKO mice presented premature ventricular complexes (PVCs), whereas PVCs were not detected in cMCM animals. These findings support the possibility that cKO hearts are vulnerable to ectopic events arising from sympathetic activation and release of catecholamines, that occur in response to the physical and emotional stress associated with the restraint method (32, 64, 65). To test this possibility, additional studies are granted.

Ex vivo experiments involving acute enhancement of *I*_NaL_ with ATX-II provide corroborating information on the ability of enhanced Na^+^ influx to interfere with diastolic function and ventricular compliance. In addition, these tests document that *I*_NaL_ has inotropic effect on the myocardium, but this aspect was not observed in mice with cardiac specific *Scn1b* deletion. Differences between ex vivo and in vivo results using our experimental models may be ascribed to dissimilarities in levels and modalities of *I*_NaL_ activation and enhancement of Na^+^ influx. Moreover, effects related to noncanonical functions of *Scn1b* may have contributed to adaptations occurring in cKO hearts. It has been recently reported that intramembrane proteolysis of the VGSC β1-subunit generates a soluble intracellular domain, which translocates to the nucleus, where it acts as a transcriptional modulator of genes involved in excitability and Ca^2+^ ion binding (4). This process, found to be operative in the hearts of juvenile mice, may remain active in adult life and may have partly contributed to the effects associated with cardiac *Scn1b* gene deletion observed here. Further studies to clarify this possibility are warranted.

The cardiac-restricted and inducible model of *Scn1b* deletion employed here has allowed us to circumvent lethality of offspring associated with complete loss of this gene (23). Moreover, it has provided a tool to evaluate the function of β1/β1B-subunits in the hearts of adult animals that have developed normally, without defective modulatory activity of the cleaved intracellular domain of the β1-subunit on gene expression (4). In relation to the use of the inducible MerCreMer system driven by the α-MHC promoter, our findings align with previous results documenting that a fraction of animals experience transient cardiotoxic effects associated with controlled gene recombination (26, 27). These results substantiate the need to introduce appropriate control animals, optimize the dose of tamoxifen administration, and identify the critical time window following treatment, in which cardiotoxicity may become manifest. Our study using both male and female mice provides details on the experimental protocol for the evaluation of the consequences of gene silencing, using models of Cre recombinase expression driven by the α-MHC promoter.

In conclusion, this study documents that VGSC β1/β1B-subunits modulate electrical and mechanical function of the heart by regulating Na^+^ influx in cardiomyocytes. These results strengthen the notion that loss of integrity of the VGSC macromolecular complex has important consequences on cardiac physiology.

## SUPPLEMENTAL DATA

10.6084/m9.figshare.19326329 Supplemental Figs S1–S15 and Supplemental Tables S1–S4: https://doi.org/10.6084/m9.figshare.19326329.

## GRANTS

This work was supported by the National Institutes of Health Grants R01AG055407 and R01HL146628, American Heart Association Grant 19TPA34850067, and New York Medical College (NYMC) intramural resources, including NYMC Translational Science Institute funds.

## DISCLOSURES

No conflicts of interest, financial or otherwise, are declared by the authors.

## AUTHOR CONTRIBUTIONS

M.R. conceived and designed research; D.O.C., E.P., H.K., S.P.P., S.T., E.C., A.C., G.V., S.J., and M.R. performed experiments; D.O.C., E.P., H.K., S.T., A.C., S.J., and M.R. analyzed data; D.O.C., E.P., S.J., J.T.J., and M.R. interpreted results of experiments; M.R. prepared figures; M.R. drafted manuscript; D.O.C., E.P., S.J., J.T.J., and M.R. edited and revised manuscript; D.O.C., E.P., H.K., S.P.P., S.T., E.C., A.C., G.V., S.J., J.T.J., and M.R. approved final version of manuscript.
